# Supplementary material for: Unveiling Drivers of Retinal Degeneration in RCS Rats: Functional, Morphological, and Molecular Insights
Source: Int J Mol Sci. 2024 Mar 28;25(7):3749. doi: 10.3390/ijms25073749 (PMC11011632; doi:10.3390/ijms25073749)
Supplement: Supplementary file 1 [file ijms-25-03749-s001.zip › Supplementary Tables S1-S3 and Figures S1-S8.pdf]

SUPPLEMENTARY TABLES AND FIGURES

Supplemental Table S1. RT-qPCR Primer Sequences

Gene	Forward (5'-3')	Reverse (5'-3')
**Cat**	TCACCTGAAGGACCCTGACA	TCCATCTGGAATCCCTCGGT
**Cyba**	GCAGGAGTGCTCATCTGTCT	GTACTTCTGTCCACACCGCTC
**Cybb (Nox2)**	TAGCACTTCACACGGCCATT	ATATGGGTCCGAAGTCCCGA
**Gapdh**	AGTGCCAGCCTCGTCTCATA	GGTAACCAGGCGTCCGATAC
**Gfap**	CAACCTCCAGATCCGAGAAACC	GCATCTCCACCGTCTTTACCA
**Nfkb1**	TCCCGCCCCTTCTAAAACTC	CTCCACCAGCTCTTTGATGGT
**Nox1**	CCTGAAGGATCCCATCAGAGA	ACCAGCCAGTTTCCCATTGT
**Nox4**	TGTTGGGCCTAGGATTGTGT	CTTCTGTGATCCGCGAAGGT
**Sod1**	AGGGCGTCATTCACTTCGAG	CCCATGCTCGCCTTCAGTTA
**Sod2**	ACCGAGGAGAAGTACCACGA	TGTGATTGATATGGCCCCCG
**Sod3**	ACACCTATGCACTCCACAGAC	AGGGGATGCTAGGGGCTTAT
**Tgfb1**	GACTCTCCACCTGCAAGACC	GGACTGGCGAGCCTTAGTTT
**Tlr4**	GATCTGAGCTTCAACCCCCT	ATTGTCTCAATTTCACACCTGGA
**Tlr7**	GCCTTCAAGAAAGATGCCATT	AGTTTGGTGGAGGAGAACAGAG
**Tlr9**	CTTCCATTTTCCATCATGGTTCTCT	GCCATGAGGCTTCAGTTCAC

Supplemental Table S2. Western Blot Antibodies

Antibody	Host	Dilution	Catalog	Manufacturer
**Cit-H3**	Rabbit	1:1000	ab5103	Abcam
**TLR4**	Mouse	1:1000	sc-293072	Santa Cruz Biotechnology
**TLR7**	Rabbit	1:1000	NBP2-24906	Novus Biologics
**TLR9**	Mouse	1:500	NBP2-24729	Novus Biologics
**GAPDH**	mouse	1:10000	60004-1-Ig	Proteintech
**Mouse Secondary**	Goat	1:10000	7076S	Cell Signaling
**Rabbit Secondary**	Goat	1:10000	7074S	Cell Signaling

Supplemental Table S3. Immunofluorescence Antibodies

Antibody	Host	Dilution	Catalog	Manufacturer
**PAD4**	Rabbit	1:100	17373-1-AP	Proteintech
**Cit-H3**	Rabbit	1:1000	ab5103	Abcam
**4-Hydroxynonenal**	Mouse	1:500	MAB3249	R&D Systems
**Malondialdehyde**	Rabbit	1:1000	ab27642	Abcam
**Mouse Secondary**	Goat	1:500	A32723	Thermo Fisher Scientific
**Rabbit Secondary**	Goat	1:500	A11037	Thermo Fisher Scientific

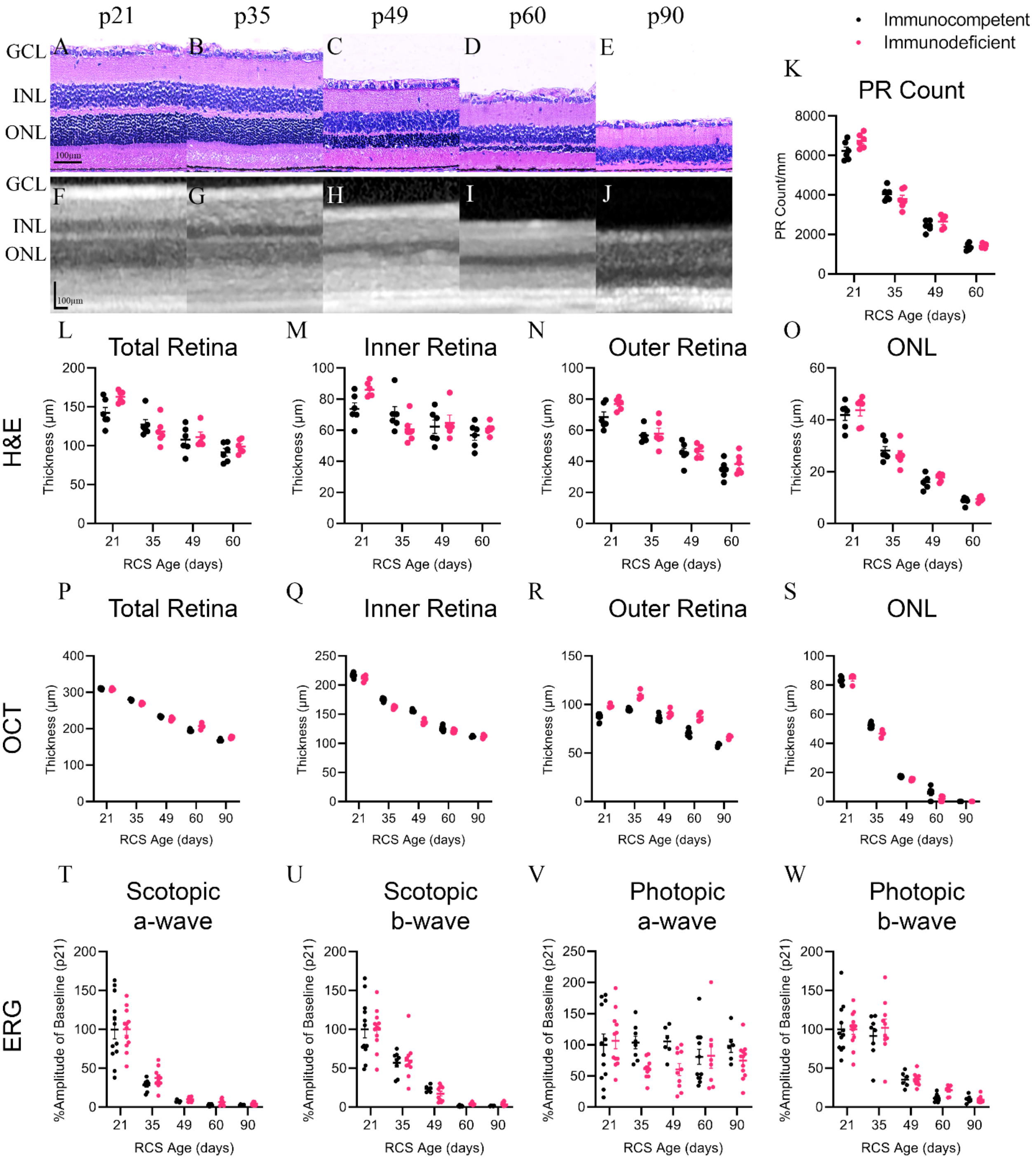

Supplemental Figure S1. Structural and function comparison of RCS and iRCS rats from p21 to p90. H&E staining and OCT imaging corroborate changes in the retina (A-J). Histology and OCT measurements are comparable between immunocompetent RCS rats (black dots) and immunodeficient RCS (red dots) at each timepoint (K-W). 3.0 cd.s/m^2 ERG amplitudes (% amplitude compared to baseline at p21) for scotopic a-and b-wave (T-U) and photopic a- and b-wave (V-W) show no significant difference between immune status at any timepoint. Data represented as mean ± SEM, two-way ANOVA with Bonferonni's correction. Histology: n=6 for each group; OCT: immunocompetent RCS n=8, 7, 3 for p21, p35-p60, and p90, respectively and immunodeficient RCS n=4 for each group; ERG: immunocompetent RCS n = 12, 8, 6, 12, 6 and immunodeficient RCS n=12, 10, 10, 8, 12 for p21, p35, p49, p60, and p90 respectively. Scale bar = 100μm.

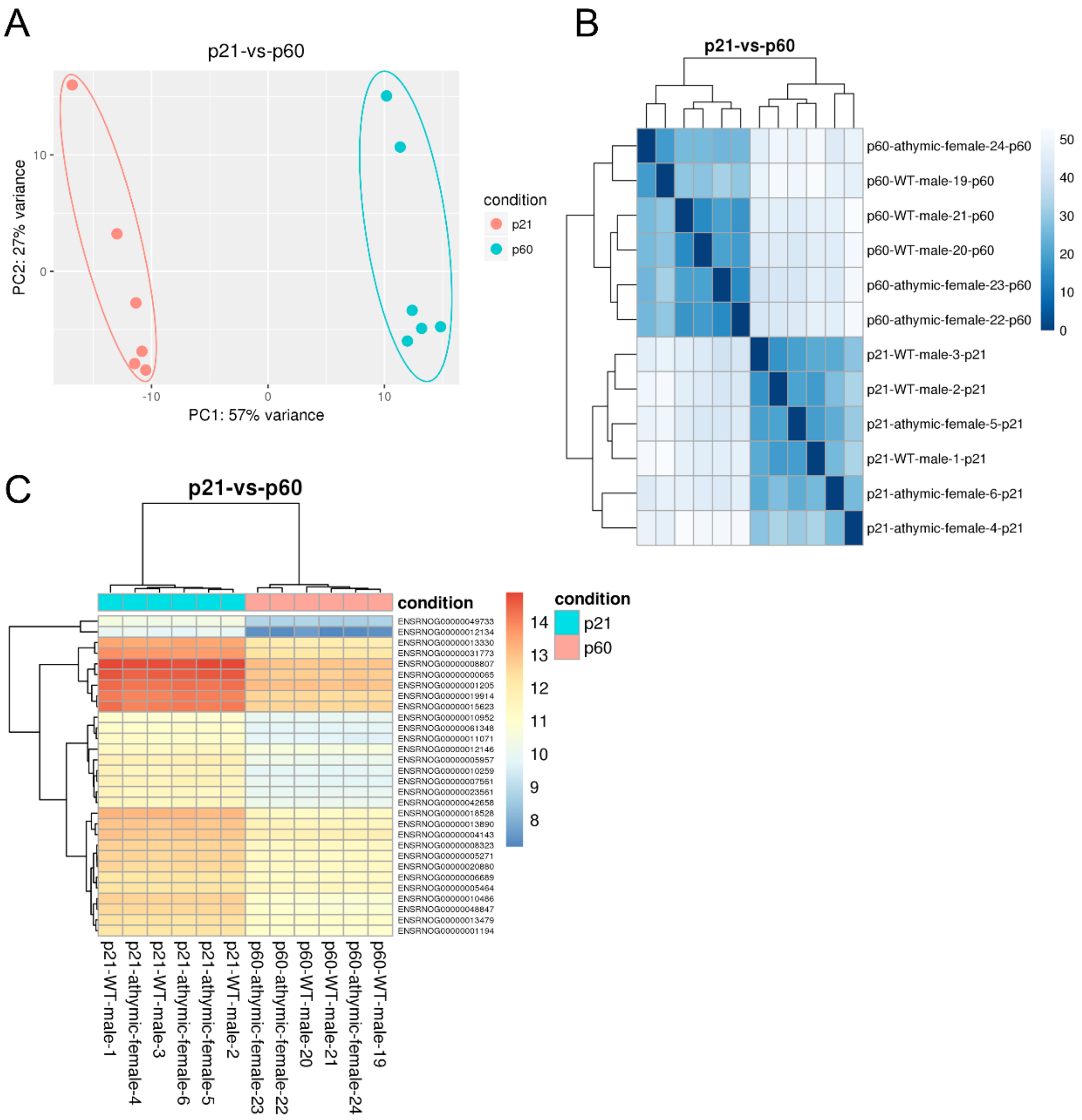

Supplemental Figure S2. Transcriptomics sample grouping shows strong age association. Analyses and graphs prepared by Azenta Life Sciences. Principal component analysis shows principal component 1 can differentiate between p21 and p60 RCS rats (A). Sample-to-sample distances shows clear differences between p21 and p60 animals (B). Biclustering of top 30 DEGs shows clear differences between p21 and p60 (C).

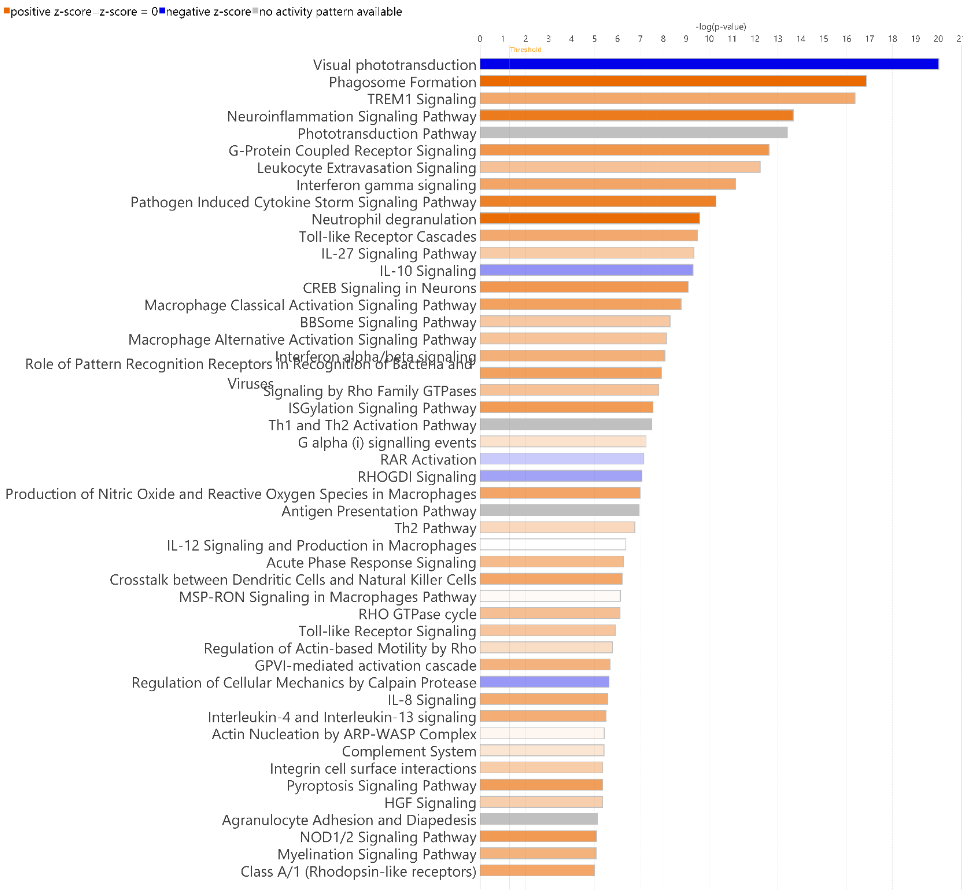

© 2000-2024 QIAGEN. All rights reserved.

Supplemental Figure S3. Top Effected Pathways in iRCS Rat. QIAGEN IPA canonical pathways of RNA-Seq using significant DEGs for p21 (n=3) vs p60 iRCS rats (n=3).

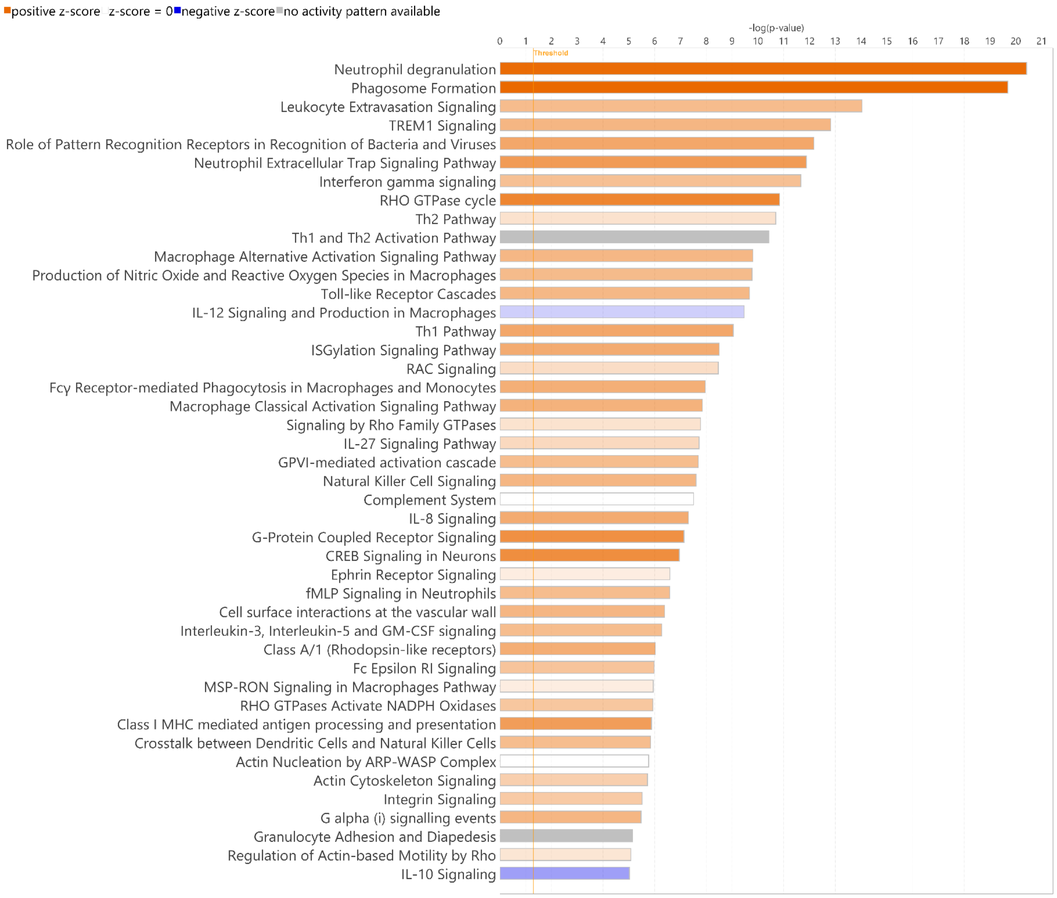

© 2000-2024 QIAGEN. All rights reserved.

Supplemental Figure S4. Top Effected Pathways in Microglia Assigned Cells of iRCS Rat. QIAGEN IPA canonical pathways of RNA-Seq using significant DEGs for p21 (n=3) vs p60 iRCS rats (n=3).

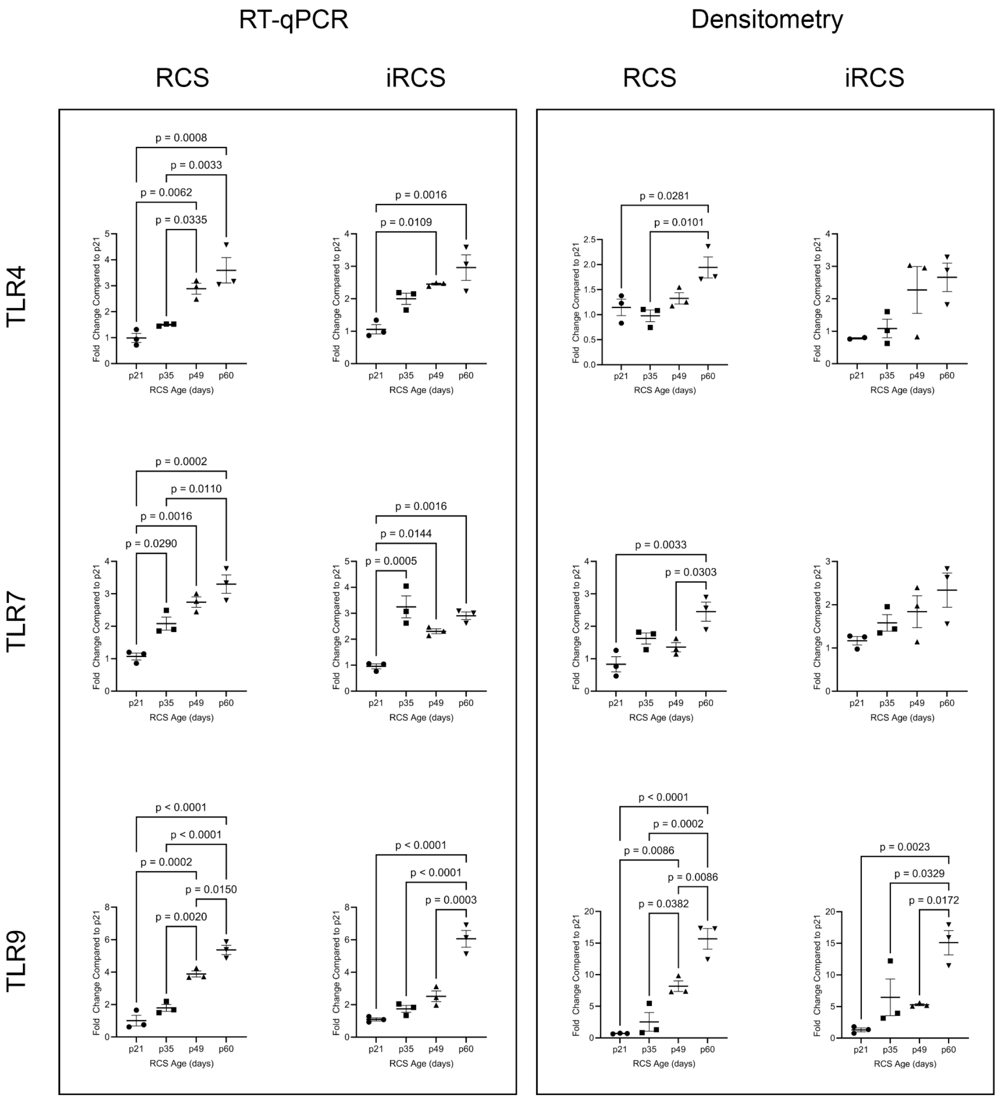

Supplemental Figure S5. TLR Expression in RCS and iRCS Retinas. Toll-like receptors (TLRs) expression was measured with RT-qPCR and immunofluorescent densitometry for RCS and iRCS at each time point p21-p60. RT-qPCR shows significant increases for all TLRs for both RCS and iRCS. iRCS TLR4 and TLR7 protein expression showed a nonsignificant trend upwards. Data represented as mean ± SEM, one-way ANOVA with Tukey's correction, n=3 each group.

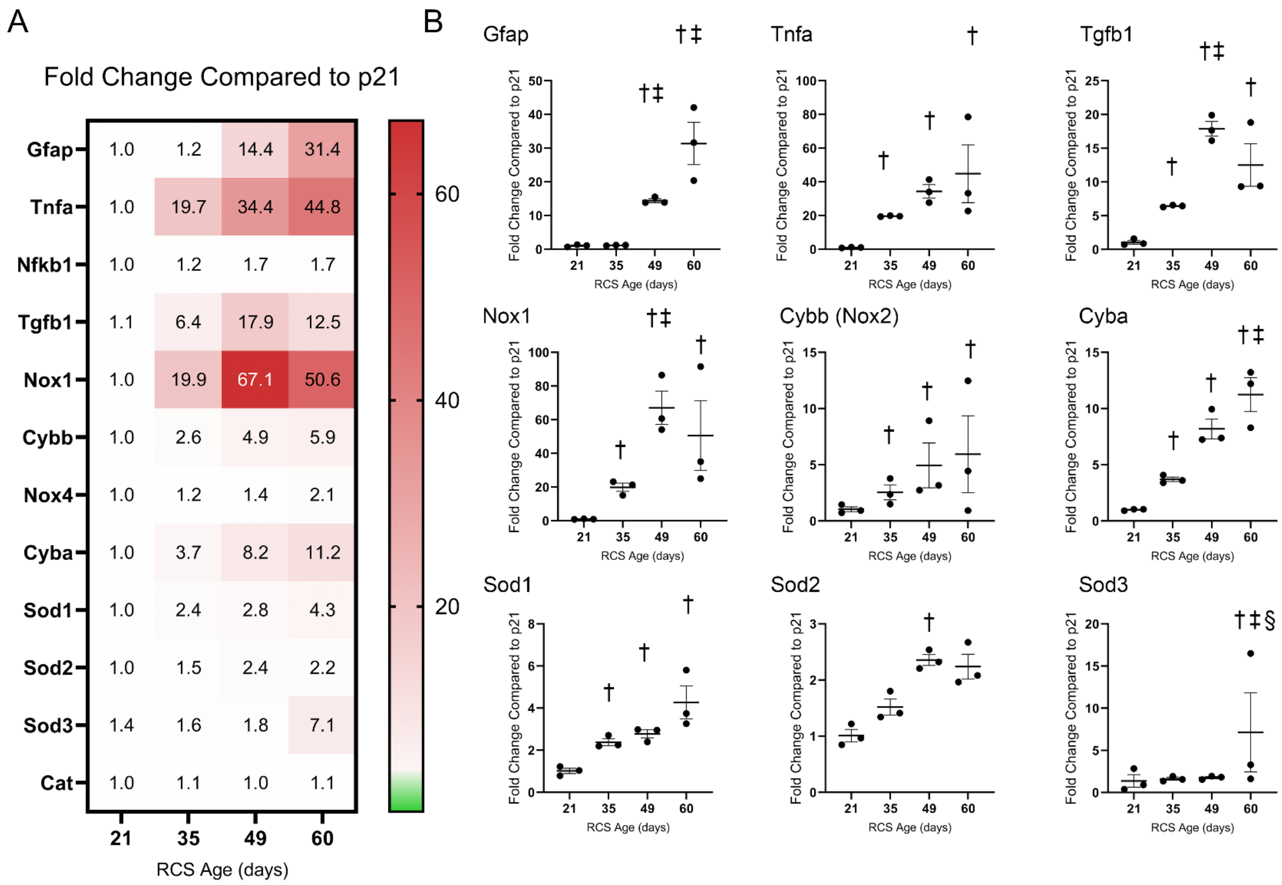

Supplemental Figure S6. Fold change of reactive gliosis, inflammation and oxidative-stress related gene expression changes compared to p21 for RCS rats. Categorical heatmap of all genes displayed with mean fold change (A). Individual dot plots of fold changes for each gene with mean ± SEM displayed (B). ΔΔCT data was analyzed using two-way ANOVA with Tukey's multiple comparison, †$p<0.05$ compared to p21, ‡$p<0.05$ compared p35, §$p<0.05$ compared to p49, n=3 each group.

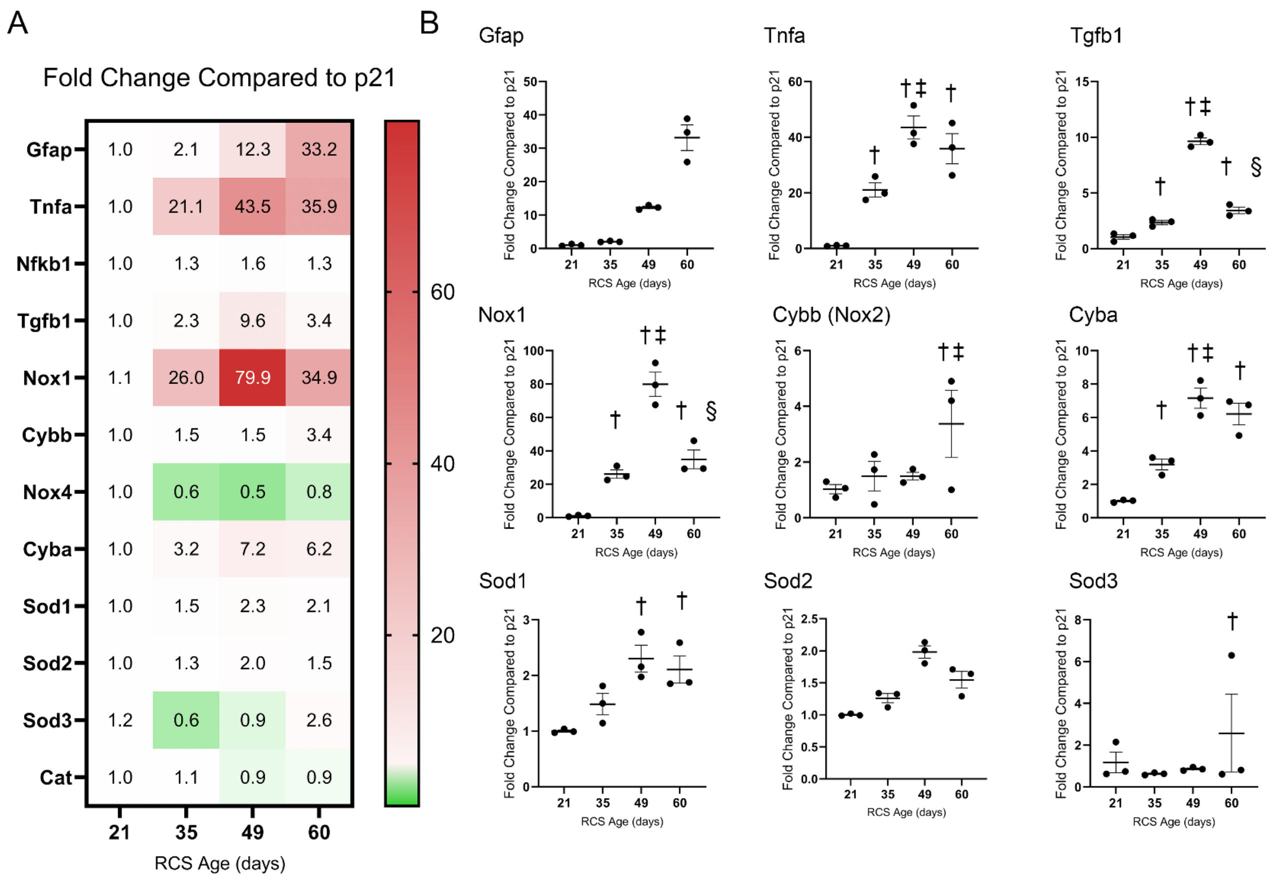

Supplemental Figure S7. Fold change of reactive gliosis, inflammation and oxidative-stress related gene expression changes compared to p21 for iRCS rats. Categorical heatmap of all genes displayed with mean fold change (A). Individual dot plots of fold changes for each gene with mean ± SEM displayed (B). ΔΔCT data was analyzed using two-way ANOVA with Tukey's multiple comparison, †p<0.05 compared to p21, ‡p<0.05 compared p35, §p<0.05 compared to p49, n=3 each group.

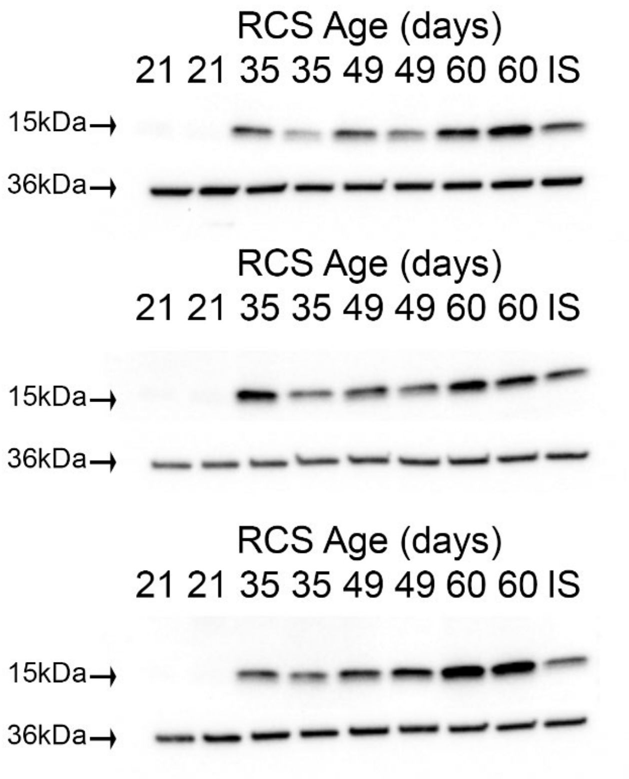

Supplemental Figure S8. Western blot analysis of RCS and iRCS retinas shows the expected band around 15kDa for CitH3 and 36kDa for GAPDH as the loading control protein. Because samples were run on multiple blots, an internal standard (IS) was used on each blot to normalize data.
